# Screening and Characterization of Phenolic Compounds from Selected Unripe Fruits and Their Antioxidant Potential

**DOI:** 10.3390/molecules29010167

**Published:** 2023-12-27

**Authors:** Akhtar Ali, Zeshan Asgher, Jeremy J. Cottrell, Frank R. Dunshea

**Affiliations:** 1 School of Agriculture, Food and Ecosystem Sciences, Faculty of Science, The University of Melbourne, Parkville, VIC 3010, Australia; akali@student.unimelb.edu.au (A.A.); zasgher@student.unimelb.edu.au (Z.A.); jcottrell@unimelb.edu.au (J.J.C.); 2 Faculty of Biological Sciences, The University of Leeds, Leeds LS2 9JT, UK

**Keywords:** unripe mango, black lemon, unripe grapes, polyphenols, flavonoids, antioxidants, human health

## Abstract

The food sector’s interest in sustainability and the demand for novel bioactive compounds are increasing. Many fruits are wasted every year before ripening due to various climatic conditions and harsh weather. Unripe mangoes, grapes, and black lemons could be rich sources of phenolic compounds that need to be fully elucidated. Using fruit waste as a source of bioactive chemicals has grown increasingly appealing as it may have significant economic benefits. Polyphenols are beneficial for human health to inhibit or minimize oxidative stress and can be used to develop functional and nutraceutical food products. In this context, this study aimed to characterize and screen unripe mangoes, grapes, and black lemons for phenolic compounds using LC-ESI-QTOF-MS/MS and their antioxidant activities. Unripe mangoes were quantified with higher total phenolic content (TPC, 58.01 ± 6.37 mg GAE/g) compared to black lemon (23.08 ± 2.28 mg GAE/g) and unripe grapes (19.42 ± 1.16 mg GAE/g). Furthermore, unripe mangoes were also measured with higher antioxidant potential than unripe grapes and black lemons. A total of 85 phenolic compounds (70 in black lemons, 49 in unripe grapes, and 68 in unripe mango) were identified, and 23 phenolic compounds were quantified using LC-MS/MS. Procyanidin B2, gallic acid, epicatechin, caffeic acid, quercetin, and chlorogenic acid were measured with higher concentration in these selected unripe fruits. A positive correlation was found between phenolic contents and the antioxidant activities of unripe fruits. Furthermore, chemometric analysis was conducted to validate the results. This study will explore the utilization of these unripe fruits to develop functional and therapeutic foods.

## 1. Introduction

Recently, the demand for natural products of superior quality has increased, which is beneficial for human health. Fruits are a rich source of vitamins, minerals, dietary fiber, and antioxidant bioactive compounds. Apple, citrus, lime, grape, banana, and mango are the most popular fruits. Globally, citrus (124.8 million metric tons (MMT)), banana (114.1 MMT), grapes (74.5 MMT), and mangoes (45.2 MMT) are produced in large quantities each year. The need for fruit production has expanded globally over the last few decades due to population growth and shifting consumer preferences for healthful diets [1]. Sustainability in food processing and waste reduction are major issues facing the modern food business [1,2]. To reduce waste and provide new sources of bioactive compounds—which are thought to act as protective agents against certain diseases like diabetes, cancer, and cardiovascular disease—researchers have concentrated on recovering the bioactive components from fruit and vegetable waste, where a significant number of phytochemicals still exist in the agro-industrial wastes [2,3,4]. Because these fruit wastes contain sizable amounts of bioactive compounds, there is currently a dearth of information regarding the use of these fruit wastes in the development of functional ingredients and food items, as well as their effects on the sensory quality of food products [2,4]. Concurrent with the increasing demand for healthier and functional foods, the food industry realized how important it was to identify natural food additives to create value-added goods with positive health effects.

Grapes (*Vitis vinifera* L). are the most prominent fruits on earth, with an annual production of over 75 million tons. The industry primarily uses grates to screen out many unripe grapes, which are then dumped into agricultural land [5,6]. Several potentially bioactive compounds, such as organic acids, phenolic compounds, vitamins, and minerals that are highly beneficial to human health, cosmetics, and biomedical areas, are thought to have been recently discovered in unripe grapes [7]. Unripe grapes are used to make verjuice and sour grape sauces, which are typically used as seasonings or acidifiers. Using immature grape waste, abundant in bioactive compounds, in agricultural areas is crucial to producing high-value foods, cosmetics, and pharmaceuticals [8,9]. Research conducted in vitro and on animals has shown that unripe grapes have anti-inflammatory, cardioprotective, anticancer, and antidiabetic qualities [9]. Recently, a renewed focus has been on the special potential applications of unripe grape extract in food and beverages, such as preservation, enrichment, or direct use in the human diet [10]. Mango (*Mangifera indica* L.) is a widely grown fruit known for its taste and nutritional value. It is a rich source of carbohydrates, vitamins, minerals, dietary fiber, and bioactive compounds like xanthonoids (mangiferin) [11,12,13]. Mangiferin is well known for its antioxidant, anticancer, antimicrobial, antiviral, antidiabetic, and antiallergic properties [14,15]. Black lemons are dried products of unripe lemons. In Middle Eastern countries these are also called dried limes. These are produced by boiling unripe limes in brine and then leaving them to dry (oven or sun drying) which gives them a dark black appearance. They are abundantly utilized in various dishes and food products to imbue a tangy flavor. Black lime tea has a unique citrusy flavor and is well-known for various health properties due to its high concentration of bioactive compounds. Black lemons are enriched with alkaloids, polyphenols, terpenes, organic acids, and vitamins [16,17].

These substances are significant because of their capacity to function as antioxidants or free-radical scavengers. This capacity is prompted by hydrogen- or electron-donating properties that are influenced by the quantity and location of hydroxyl groups in the compounds’ structure [18,19,20]. They are effective as antidiabetic, anticancer, antihypertensive, anti-inflammatory, antiaging, cardioprotective, and neuroprotective medicines because of their antioxidant qualities [18,19,20]. From this vantage point, much research has been conducted on the fortification of fruit wastes in extruded snacks, short-dough biscuits, yoghurt, kefir, and functional cookies to create unique functional food products [1]. Therefore, it is vital to recover phenolic compounds from wasted unripe fruits, particularly when searching for sustainable and affordable bioactive compounds that could be added to different food matrices to increase their nutritional value and used as a natural food coloring [21]. Even though the food sector produces enormous volumes of trash, seasonal production and the changeable composition of waste products provide a significant barrier to its industrial use.

So, using fruit waste as a source of bioactive chemicals has grown increasingly appealing as it may have significant economic benefits [4,21]. To extract value-added byproducts from agro-industrial wastes, identifying bioactive components—specifically, phenolics—has drawn interest [22]. There is a wealth of information regarding the efficacy of phenolic compounds as natural antioxidants and their ability to prevent disease, making them one of the most significant categories of natural antioxidants of interest [23,24]. Several studies have been conducted on industrial waste, but studies on unripe fruit waste are scarce. Therefore, this study aimed to screen out the selected sour fruit waste with high antioxidant activities, which could function as readily available and affordable sources of bioactive compounds for use in the food and pharmaceutical industries. This study aimed to identify and quantify phenolic compounds and their antioxidant capacity in unripe grapes, mangoes, and black lemons using state-of-the-art analytical tools, including LC-ESI-QTOF-MS/MS. We measured total phenolic contents (TPC), total flavonoid contents (TFC), and total condensed tannins (TCT). At the same time, antioxidant activities, including 2,2-azinobis-3-ethylbenzothiazoline-6-sulfonic acid (ABTS), 2,2-diphenyl-1-picryl-hydrazyl-hydrate radical scavenging activity (DPPH), hydroxyl radical scavenging assay (OH-RSA), and ferrous ion chelating activity (FICA), were also analyzed. Chemometric analysis further validates the results of this study. This study provides evidence that these wasted fruits have the potential to be used in pharmaceuticals, cosmetics, nutraceuticals, and food sectors.

## 2. Results and Discussion

### 2.1. Measurement of Phenolic Contents of Unripe Fruits

The distinct qualities and advantageous potential of phenolic compounds, such as their anti-inflammatory, antioxidant, antidiabetic, antimicrobial, and anticarcinogenic qualities, have drawn the attention of scientists [25]. Table 1 displays black lemons’, unripe mangoes’, and unripe grapes’ phenolic content and antioxidant examinations. Reports have shown that sour fruits are high in phenolic compounds [23]. TPC, TFC, and TTC were used in this study to measure the phenolic content of the three sour fruits, namely black lemon, unripe mango, and unripe grapes (Table 1). Unripe mangoes had the highest phenolic content of all the samples since they had significantly greater TPC and TFC and higher TCT (*p* < 0.05) than the other samples.

The Folin–Ciocalteu method was used to determine total phenolic contents [26]. Every sample differed considerably (*p* < 0.05) in terms of TPC. The unripe mango (58.01 ± 6.37 mg GAE/g) had the highest content of phenolic compounds, with black lemon (23.08 ± 2.28 mg GAE/g) following closely behind. Among all samples, unripe grapes showed the lowest TPC value of 19.42 ± 1.16 mg GAE/g. The variance in fruit maturity levels used in this study and subsequent investigations for measuring phenolic contents may have contributed to the differences in TPC results. In a previous study by Arshad Mehmood et al. [27] the TPC of ripe mango cultivars assessed in the peel and pulp samples showed that the peel samples had a higher TPC than the pulp. The TPC results of unripe mango samples are comparable to those of reports for the TPC (62.45 d ± 1.25 mg GAE/g) and (51.68 ± 0.66 mg GAE/g) of mango pulp of the F2-Narcissus mango and F9-Egg mango, respectively [27]. Previously, Dorta et al. [28] measured TPC between 35 and 98 mg GAE/g in the peel of various mango cultivars in Spain. This variation in the concentration of polyphenols was observed in different studies [12].

Black lemon (16.41 ± 1.02 mg GAE/g) and unripe grapes (15.01 ± 1.12 mg GAE/g) did not substantially differ from one another in TFC, while unripe mango (44.94 ± 5.02 mg QE/g) did demonstrate a significantly (*p* < 0.05) higher concentration above the others. On the other hand, unripe mango was found to have a considerably higher phenolic compound profile, which makes it a beneficial natural antioxidant or functional ingredient in food preparation. The previous study provided estimated values of the total flavonoid content in the examined mango samples and found that the peel samples had a much higher level of TFC than the pulp samples, which is opposite to their results for TPC. The TFC value of the unripe mango in our study is consistent with the peeled mango variety, F2-Narcissus mango (48.87 ± 1.50) [27].

In the case of TCT, at 11.41 ± 0.91, the unripe mango cultivar had the highest total tannin content. In contrast, unripe grapes and black lemon cultivars come after it with a nominal difference among their TCT values, as 4.28 ± 0.62 and 4.01 ± 0.32, respectively. In a previous study, the highest concentration of tannins was found in lemon (7.4 ± 0.14 mg tannic acid equivalent (TAE)), with citrus lemon leaf extract coming in second (5.9 ± 0.20 mg TAE). The least amount of tannins (4.8 ± 0.18 mg TAE) is found in citrus lemon root, comparable to our study results [29].

Flavonoids are an abundant class of polyphenols; they are vital secondary metabolites in fruits, herbs, and vegetables, having a positive impact on human health. Previously, no single study was conducted on these unripe fruits in such a comprehensive way. This study will explore the therapeutic and functional utilization of these unripe fruits in the food industry.

### 2.2. Antioxidant Potential of Unripe Fruits

Antioxidants are essential components of many products that serve as metal chelators, radical oxygen scavengers, reducing agents, and H^+^ ion donors. The antioxidant capacity of unripe mango, black lemon extracts, and unripe grapes was assessed using a variety of assays, such as DPPH, ABTS, ferrous ion chelating activity (FICA), and OH-RSA. Table 2 shows the antioxidant potential results for the three samples (unripe mango, unripe grapes, and black lemon).

Sour fruits are included in the human diet as essential sources of antioxidant compounds capable of preventing free radical damage. The main active antioxidants in these fruits are generally phenolic compounds, which are thought to provide a variety of health advantages. These substances are multipurpose agents that work in biological systems as hydrogen ion donors, strong reducers, metal chelators, and scavengers of free radicals [4]. The exceptional antioxidant capacity of sour fruits is crucial for evaluating their dietary benefits. Numerous components found in these fruits are known to have antioxidant qualities. Many bioactive substances, primarily phenolic compounds, are potential therapeutic agents for reducing the harmful impacts of free radicals. These compounds include flavonoids, tannins, procyanidins, coumarins, phenolic acids, stilbenes, xanthones, lignans, and other polyphenols [30].

Antioxidants possess the ability to either inhibit or stop other molecules from oxidizing. An approach for determining antioxidant capacity is the DPPH radical scavenging assay, which measures a decrease in absorbance following oxidation processes [31]. Antioxidants can retard color loss which has allowed scientists to calculate the scavenging activity [31]. The DPPH values of unripe mango (114.27 ± 9.42 mg AAE/g) are estimated to be significantly higher, at a *p* < 0.05, than the two listed sour fruits. The research results demonstrated a significant difference between the DPPH values of black lemon (32.53 ± 2.47 mg AAE/g) and unripe grapes samples (23.71 ± 2.17 mg AAE/g) (Table 2). The significant differences in the DPPH results between our study and the earlier study could have several causes, particularly regarding unripe mango. The fruit samples used in our study may have come from different sources and are of higher quality than those used in the previous research, which could substantially affect their antioxidant content [32]. Our prior study addressed the mango peel and pulp separately [32], while our sample in this study is an entire unripe mango. The amount of antioxidants in the fruit may vary depending on its parts [33]. The second reason might be that the antioxidant extraction process of the fruit samples had a significant impact on the outcomes [33]. Different concentrations of antioxidants in the extracts can result from variations in extraction conditions, solvents, and efficiency [34].

Most people agree that the ABTS assay is the most affordable. This is such that, in comparison to the other assays, the ABTS assay can afford to produce its synthetic chromogenic substrate [35]. For assessing the antioxidant potential of a range of materials, including foods, dietary supplements, and biological samples, the ABTS (2,20-azino-bis(3-ethylbenzothiazoline-6-sulfonic acid)) assay is regarded as a versatile and efficient technique [36]. Since the ABTS assay relies on the interaction of ABTS with an agent that exhibits antioxidant activity, it is also comparatively easy to conduct [37]. According to the results of the ABTS assay, unripe mango (134.12 ± 9.63 mg AAE/g) exhibited significantly (*p* < 0.05) higher activities than black lemon (69.25 ± 5.17 mg AAE/g) and unripe grapes (53.44 ± 4.79 mg AAE/g). In a previous study by Deepa Madalageri et al., mango peel had a significantly higher mean total antioxidant activity (24.782 mg TE/g DM or 99.128 μmol TE/g DM) than mango pulp (1.964 mg TE/g DM or 7.856 μmol TE/g DM) when the means of the ABTS assay were compared [38]. Le (2012) reported that the dehydrated mango’s ABTS scavenging activity ranged from 46.7 to 73.8 μmol TE/g DM [39]. Using the ABTS assay, dried mango pulp’s antioxidant activity was 27.1 μmol/g db ascorbic acid equivalent [40]. According to Sogi et al. (2014), the antioxidant properties of dried mango samples ranged from 50.7 to 103.8 μmol TE/g db [41].

A ferrous ion chelating assay was also performed to assess the antioxidant potential of the selected sample [42]. The results indicated that, compared to black lemon (14.24 ± 1.07 mg EDTA/g) and unripe grapes (11.49 ± 0.87 mg EDTA/g), unripe mango had a significantly (*p* < 0.05) higher FICA potential (33.16 ± 2.08 mg EDTA/g). Furthermore, the scavenging activity of OH radicals was conducted to estimate the antioxidant profile. The results showed that unripe mangoes had the highest FICA (57.31 ± 4.85 mg AAE/g) while unripe grapes had the lowest FICA (19.57 ± 1.42 mg AAE/g).

### 2.3. Pearson Correlation

Pearson’s correlation was conducted between phenolic contents and their antioxidant activities. The results of Pearson’s correlation analyses are presented in Table 3.

**Figure 1:**
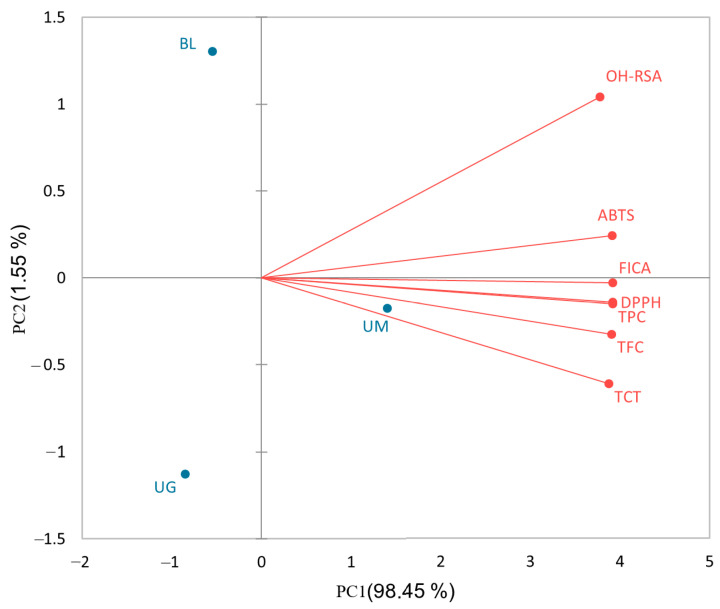
Biplot analysis of phenolic contents and antioxidant activities of sour fruits (black lemon (BL), unripe mangoes (UM), and unripe grapes (UG)).

The results show that TPC and TFC have very strong correlations (*p* < 0.05), indicating that flavonoids are the main class of phenolic compounds in selected unripe sour fruits. TFC and TCT are also significantly correlated, which means that flavonoids, particularly condensed tannins, are very potent in these sour fruits. Overall, TPC and TFC are highly correlated with antioxidant activities (DPPH and FICA). The results are supported by previous studies where it has been reported that total phenolics and flavonoids are the main compounds responsible for the antioxidant capacity of fruits, vegetables, and herbs [25,43,44]. Moreover, a biplot analysis (Figure 1) was conducted to further understand the contribution of selected sour fruits in phenolic contents and their antioxidant activities.

The biplot indicates that PC1 (principal component 1) shares a 98.5% contribution in this study, showing that unripe mango has the highest concentration of phenolic compounds and strong antioxidant activities. The phenolic contents (TPC, TFC, and TCT) are positively correlated with antioxidant activities (DPPH, FICA, ABTS, and OH-RSA).

### 2.4. LC-MS/MS Analysis

An untargeted analysis was conducted to screen and characterize phenolic compounds in complex unripe mangoes, grapes, and black lemon extracts using LC-ESI-QTOF-MS/MS. Only the compounds with less than 10 ppm error and more than 80 PCDL scores were reported in this study (Table 4).

#### 2.4.1. Flavonoids

Flavonoids are the most abundant class of phenolic compounds having strong antioxidant, free-radical-scavenging properties [45]. Thirty-five flavonoids were detected in the selected plants. Compound **3** (cyanidin, *m*/*z* 287) was seen in the positive mode in all three samples. Compounds **1** and **2** were tentatively identified as cyanidin 3-rhamnoside 5-glucoside and petunidin 3-rhamnoside 5-glucoside in a positive mode in the black lemon sample, which made product ions at *m*/*z* 449 and 287, and *m*/*z* 480 and 317, respectively. Compounds **1**, **2**, and **3** were characterized as anthocyanins. Procyanidin trimmer C1 (compound **6**) and (−)-epigallocatechin 7-*O*-glucuronide (compound **8**) at ESI^+^ *m*/*z* 867.2131 and *m*/*z* 483.1133 produced fragment ions at *m*/*z* 867 and 483, respectively. Three more compounds, epicatechin (compound **4**, *m*/*z* 289.0717), procyanidin B2 (compound **5**, *m*/*z* 577.1351), and procyanidin dimmer B2 (compound **7**, *m*/*z* 579.1497) were detected in unripe mango, unripe grapes, and black lemon, which made product ions at *m*/*z* 245 and 205; *m*/*z* 451, 425, 407, and 289; and *m*/*z* 579. Procyanidin trimmer C1 and procyanidin dimmer B2 have previously been found in native Australian flora [46,47]. 6-Geranylnaringenin (compound **10**) was found in black lemon by displaying the product ions at *m*/*z* 287, 243, 159, and 119 at ESI^−^ *m*/*z* 407.1864. Compound **9** (didymin) and compound **11** (hesperidin) were detected in unripe mango, unripe grapes, and black lemon and produced fragment ions at *m*/*z* 577 and 287 and *m*/*z* 303. Hesperidin and hesperetin were previously detected and measured by Velamuri et al. [48] and Sharma et al. [49] in sage (*Salvia officinalis*) and rosemary (*Rosmarinus officinalis*). Compound **12**, identified as hesperetin 3′-*O*-glucuronide (*m*/*z* 479.1184) with product ions at *m*/*z* 301, was found in unripe grapes with the positive ionization mode. Hesperetin-3-*O*-glucuronide was previously discovered in the Exocarpium Citri grandis (ECG) extract, according to Zeng et al. [50]. Nobiletin (compound **13**), apigenin 6-C-glucoside (compound **14**), and diosmin (compound **18**) at ESI^−^ were putatively identified in unripe mango, unripe grapes, and black lemon, which produced daughter ions at *m*/*z* 401, *m*/*z* 269, and *m*/*z* 301, respectively. One of the most well-known characteristics of the flavonoid nobiletin is its neuroprotective properties [51,52]. Research has also investigated its antioxidant, anti-inflammatory, and antiobesity properties, making it a promising natural substance for various medical applications [52]. Compound **16** (*m*/*z* 595.1658) and compound **17** (*m*/*z* 449.1079) at ESI^+^ produced fragment ions at *m*/*z* 595, 303, and 285. Compound **15** was tentatively identified as apigenin 7-*O*-glucoside with product ions at *m*/*z* 417 and was found in unripe grapes and unripe mangoes samples in the negative ionization mode.

Kaempferol 3,7,4′-*O*-triglucoside (*m*/*z* 773.2135), isorhamnetin 3-*O*-glucuronide (*m*/*z* 493.0977), and kaempferol 7-*O*-glucoside (*m*/*z* 448.1000) were recognized as the compounds **19**, **20**, and **21**, and each of them displayed the product ions at *m*/*z* 773, 493, and 448; they existed in unripe mango and black lemon sample, respectively, with the positive ionization mode. Myricetin (compound **22**) and quercetin (compound **26**) at ESI^+^ *m*/*z* 319.0449 and *m*/*z* 303.0499, respectively, which were tentatively identified in all three samples and generated product ions at *m*/*z* 319 and *m*/*z* 285 and 169, respectively, were detected in MS/MS. In a previous study, a quercetin compound with *m*/*z* 301.0332 was identified in mint, basil, and fenugreek leaves [10]. Dihydroquercetin (compound **23**) and kaempferol 3-*O*-glucuronide (compound **24**) produced fragment ions at *m*/*z* 305 and 463, respectively. Dihydromyricetin 3-*O*-rhamnoside (*m*/*z* 467.1184) was observed as compound **25** in the positive mode and produced product ions at *m*/*z* 467 that were identified in the black lemon and unripe grape sample. Dihydromyricetin 3-*O*-rhamnoside is unique in that it can lessen the harmful effects of alcohol consumption and enhance liver function. This distinctive substance is widely recognized for its hepatoprotective properties. Four more compounds, 6″-*O*-acetylglycitin (compound **27**), 6″-*O*-acetylgenistin (compound **28**), 3′-*O*-methylequol (compound **32**), and dihydrobiochanin A (compound **33**) in the positive ionization mode produced fragment ions at *m*/*z* 489, 475, 273, 269, 203, 201, and 175, respectively. Compound **30** (violanone) and compound **31** (dihydroformononetin) were detected in black lemon, unripe grape, and mango [40]. Compound **29** (4′,7-dihydroxyisoflavan) at ESI^−^ *m*/*z* 241.0870 produced fragment ions at *m*/*z* 241. Previously, it had been identified in mint, rosemary, sage, basil, and oregano [3]. Compound **34**, identified as phloridzin (*m*/*z* 435.1297) with product ions at *m*/*z* 435, was found in black lemon and unripe mango with the negative ionization mode. Phloretin 2′-*O*-glucuronide (compound **35**) at ESI^−^ *m*/*z* 451.1235 generated a product ion at *m*/*z* 275.

#### 2.4.2. Stilbenes and Lignans

The health benefits of stilbenes and lignans make them essential phenolic compounds. In this study, we reportedly discovered nine phenolic metabolites in various therapeutic sour fruits. 4′-hydroxy-3,4,5-trimethoxystilbene (compound **36**) and resveratrol (compound **39**) at ESI^+^ *m*/*z* 287.1278 and 229.0859, respectively, generated a product ion at *m*/*z* 287, 211, 167, and 127 and were found in unripe mango and unripe grape. Dihydroresveratrol and piceatannol were identified in unripe mango and black lemon by displaying product ions at *m*/*z* 229.0872 and 245.0823 in the positive ionization mode. Compound **37** (piceatannol 3-*O*-glucoside and *m*/*z* 405.1191) produced a product ion at *m*/*z* 245 and was identified in all three samples in the negative ionization mode. Compound **41** (7-hydroxymatairesinol) was identified with the positive ionization mode in unripe mango and black lemon. Lu and Yeap Foo [44] also reported the antioxidant activity of sagerinic acid. Sagerinic acid is widely distributed in herbs and spices. Compounds **42** (schisantherin A) and **46** (schisanhenol) at ESI^+^ *m*/*z* 535.1973 and 401.1969 were tentatively identified with the negative ionization mode in unripe mango and black lemon. Lariciresinol (compound **43**) and 2-hydroxyenterodiol (compound **45**) formed product ions at *m*/*z* 329, 192, 178, and 175 and *m*/*z* 299, 287, 269, and 257, respectively. 2-Hydroxyenterolactone (*m*/*z* 313.1081) and secoisolariciresinol (*m*/*z* 361.1656) were recognized as the compounds **44** and **47**, and each of them displayed product ions at *m*/*z* 255 and 346, 315, 223, and 165, which existed in unripe grapes, unripe mango, and black lemon, respectively. Previously, whole-grain rye bran was discovered as containing secoisolariciresinol, 7-hydroxymatairesinol, and lariciresino through MS/MS analysis, as Hanhineva et al. [11] reported.

#### 2.4.3. Phenolic Acids

Twenty-four compounds were recognized as phenolic acids. Phenolic acids are said to have greater sensitivity in the negative mode. Compounds **48**, **49**, **50**, **53**, **55**, **56**, and **57** were putatively characterized as protocatechuic acid 4-*O*-glucoside (ESI^−^ *m*/*z* 153), gallic acid (ESI^−^ *m*/*z* 125), ellagic acid (ESI^−^ *m*/*z* 284, 257), protocatechuic acid (ESI^−^ *m*/*z* 109), *p*-hydroxybenzoic acid (ESI^−^ *m*/*z* 93), syringic acid (ESI^−^ *m*/*z* 182, 153), and benzoic acid (ESI^−^ *m*/*z* 103, 93); each were identified in black lemon, unripe grapes, and unripe mangoes, confirmed through the MS/MS pure standard. Serrano et al. reported species (Salviinae, Mentheae, and Lamiaceae) containing protocatechuic acid [12]. Previously, *p*-coumaric acid hexoside, ferulic acid, gallic acid, and chlorogenic acid were reported by Palafox-Carlos et al. [53]. Syringic acid lowers blood pressure, reduces the risk of blood clots, and raises lipid levels, all of which may benefit heart health. It may also provide health benefits such as anti-inflammatory and antioxidant properties. Syringic acid may also have antiviral, antibacterial, and anticancer effects and exhibits potential use as a natural food preservative [54]. 3-*O*-Methylgallic acid (compound **51**) and gallic acid 3-monogallate (compound **54**) were identified in black lemon and unripe mangoes at *m*/*z* 183.0299 and 323.0398, confirmed through the MS/MS product ion at *m*/*z* 169 and 125 in the negative ionization mode. Furthermore, paeoniflorin (compound **52**) produced fragments at ESI^+^ *m*/*z* 481.

Caffeic acid 4-*O*-glucuronide, chlorogenic acid, *p*-coumaric acid, caffeic acid, cinnamic acid, and ferulic acid at ESI^−^ were putatively identified in unripe mango, unripe grapes, and black lemon, which produced daughter ions at *m*/*z* 179; *m*/*z* 191, 179, and 161; and *m*/*z* 119, *m*/*z* 135, *m*/*z* 103, *m*/*z* 178, 149, and 134, respectively. Prior research on native Australian flora revealed the presence of *p*-coumaric acid, caffeic acid, cinnamic acid, and ferulic acid, all of which have strong antioxidant potential [46,47]. Caffeic acid has antibacterial, anti-inflammatory, and antioxidant properties. It also has anticancer and antidiabetic effects. Numerous potential health advantages of cinnamic acid include its antibacterial, antidiabetic, anticancer, anti-inflammatory, and antioxidant properties [55]. According to some research, cinnamic acid helps protect skin from sunburn by absorbing UV rays such that it may be helpful as a natural sunblock ingredient [55,56,57]. Ferulic acid has a wide range of known medical benefits, including anticancer, antioxidant, antidiabetic, antiaging, radioprotective, hypotensive, neuroprotective, antiatherogenic, and pulmonary protective effects [58,59]. Compounds **58** (cinnamoyl glucose—C_15_H_18_O_7_) and **62** (*p*-coumaric acid 4-*O*-glucoside—C_15_H_18_O_8_) were putatively identified in black lemon and unripe mango in the negative mode at *m*/*z* 311 and *m*/*z* 163. Sinapic acid (compound **68**) was confirmed through the pure standard at *m*/*z* 223.0612 in unripe mango and black lemon. Furthermore, rosmarinic acid (compound **70**) was detected only in black lemon, producing product ions at *m*/*z* 197 and 179. Compound **65** (dihydroferulic acid, *m*/*z* 197.0809), compound **67** (1,2,2′-triferuloylgentiobiose, *m*/*z* 871.2655), compound **69** (verbascoside A, *m*/*z* 669.2389) and compound **71** (*p*-coumaroyl glycolic acid, *m*/*z* 223.0601) were identified in the positive mode of ionization after producing a product ion at ESI^+^ *m*/*z* 197, 871, 669, and 223, respectively. Rosmarinic acids have antibacterial, anti-inflammatory, antioxidant, and antidiabetic qualities that may benefit human health. Rosmarinic acid may be beneficial as a natural allergy treatment because it reduces airway inflammation and improves respiratory function [60,61,62].

#### 2.4.4. Other Compounds

*p*-Anisaldehyde (compound **80**) and scopoletin (compound **85**) were detected in black lemon, unripe grapes, and unripe mangoes in the positive ionization mode. Compound **72** (*m*/*z* 319.1187) and compound **75** (*m*/*z* 191.0561) were identified as 3,4-DHPEA-EDA and quinic acid after producing a product ion at ESI^−^ *m*/*z* 275 and 195 and *m*/*z* 171, 127, and 85, respectively. Mangiferin, as well as mangiferin 6′-gallate, produced a characteristic fragment at *m*/*z* 331, 301, 259, and 421, and each was observed to be found in unripe mango. Previously, it was also identified and quantified in oregano, rosemary, mint, basil, bay, and thyme. Furthermore, *p*-HPEA-AC (compound **74**) and mellein (compound **79**) were detected in black lemon and unripe grapes, while each compound produced product ions at *m*/*z* 137, 119, and 133. Compounds **83** and **84** (coumarin and 4-hydroxycoumarin) generated fragment ions at ESI^+^ *m*/*z* 103 and 91 and *m*/*z* 163 in black lemon and unripe mango. Three more compounds, pyrogallol (compound **73**), carvacrol (compound **81**), and esculetin (compound **82**), form product ions at *m*/*z* 127, *m*/*z* 151, and *m*/*z* 179, respectively.

### 2.5. Venn Distribution of Phenolic Compounds in Unripe Fruits

**Figure 2:**
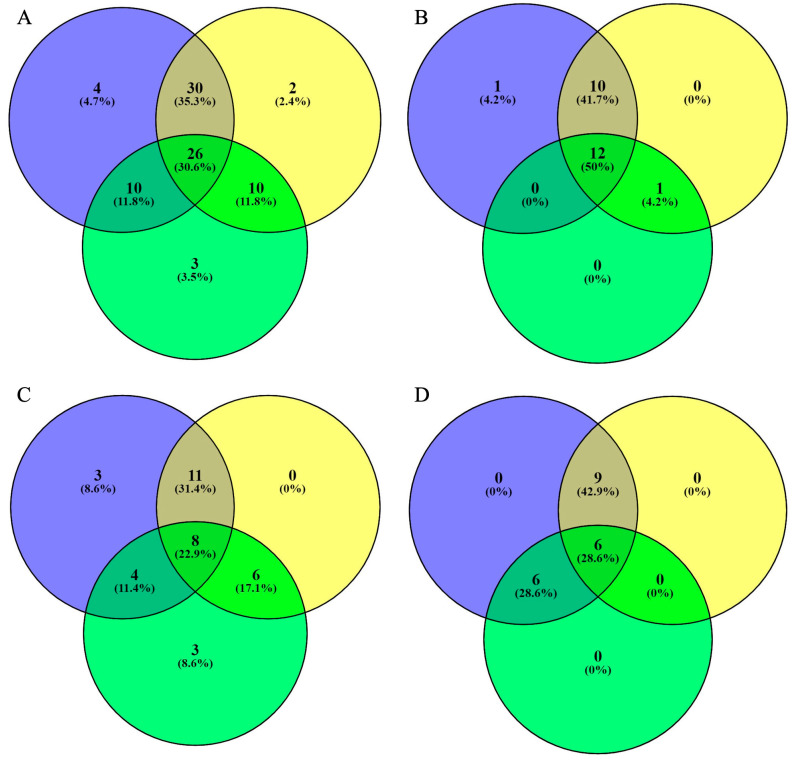
Venn distribution of phenolic compounds (total number of phenolic compounds (**A**), total number of phenolic acids (**B**), total number of flavonoids (**C**), and total number of other compounds (**D**)) in black lemon (blue), unripe mango (yellow), and unripe grapes (green).

The results are presented as a Venn distribution in Figure 2. Figure 2A shows the total number of phenolic compounds in black lemon (blue), unripe mangoes (yellow), and unripe grapes (green). It indicates that black lemon contains the highest number of unique phenolic compounds (four; 4.7%), while unripe mangoes and grapes contain two and three unique phenolic compounds, respectively. A total of 26 (30.6%) phenolic compounds overlapped in all three sour fruits, while 30 (35.3%) compounds overlapped in black lemon and unripe mangoes. Figure 2B displays the total number of phenolic acids in unripe mangoes, grapes, and black lemons. A total of 12 (50%) phenolic acids overlapped in all three fruits, while a total of 10 (41.7%) compounds overlapped in black lemon and unripe mangoes. Figure 2C shows the total number of flavonoids in the selected sour fruit waste. Three unique flavonoids were identified in each black lemon and unripe grape. A total of 11 (31.4%) flavonoids overlapped in black lemon and unripe mangoes, while a total of 8 (22.9%) flavonoids overlapped in all three fruits. Figure 2D depicts the total number of other compounds in the selected fruit waste. A total of nine (42.9%) compounds overlapped in black lemon and unripe mangoes, while a total of six (28.6%) compounds overlapped in all three fruits’ waste. Overall, unripe mangoes, grapes, and black lemons contain diverse compounds with therapeutic and pharmaceutical properties.

### 2.6. Heatmap Clustering of Quantified Phenolic Compounds in Unripe Fruits

The results of quantified phenolic compounds in unripe mangoes, grapes, and black lemons are presented in Table 5. A total of ten phenolic acids were quantified in the selected unripe fruits. Gallic acid (221.17 ± 9.69 μg/g), caffeic acid (177.31 ± 5.01 μg/g), chlorogenic acid (115.98 ± 8.79 μg/g), and ellagic acid (113.93 ± 8.31 μg/g) are the most abundant phenolic acids in unripe grapes and in unripe mangoes while ellagic acid (134.91 ± 11.42 μg/g) and syringic acid (113.42 ± 9.01 μg/g) are quantified in higher concentrations in black lemons. Chlorogenic acid, ellagic acid, and caffeic acid are abundant in unripe grapes. A total of eight flavonoids and four other compounds were quantified in the selected unripe fruits. Epicatechin (224.31 ± 13.41 μg/g) was quantified in higher concentration in unripe mangoes while the lowest concentration of epicatechin (88.52 ± 6.02 μg/g) was quantified in unripe grapes. Procyanidin B2 is a condensed tannin whose concentration was higher in black lemons (224.15 ± 11.34 μg/g) while the lowest concentration of procyanidin B2 was measured in unripe grapes (131.18 ± 10.06 μg/g). A small concentration of mangiferin was quantified only in unripe mangoes (44.93 ± 3.67 μg/g). Previously, mangiferin was measured in higher concentration in the peel (221 μg/g) than in the pulp (32 μg/g) [63]. The variation in concentration of polyphenols has been observed in different studies [12].

**Figure 3:**
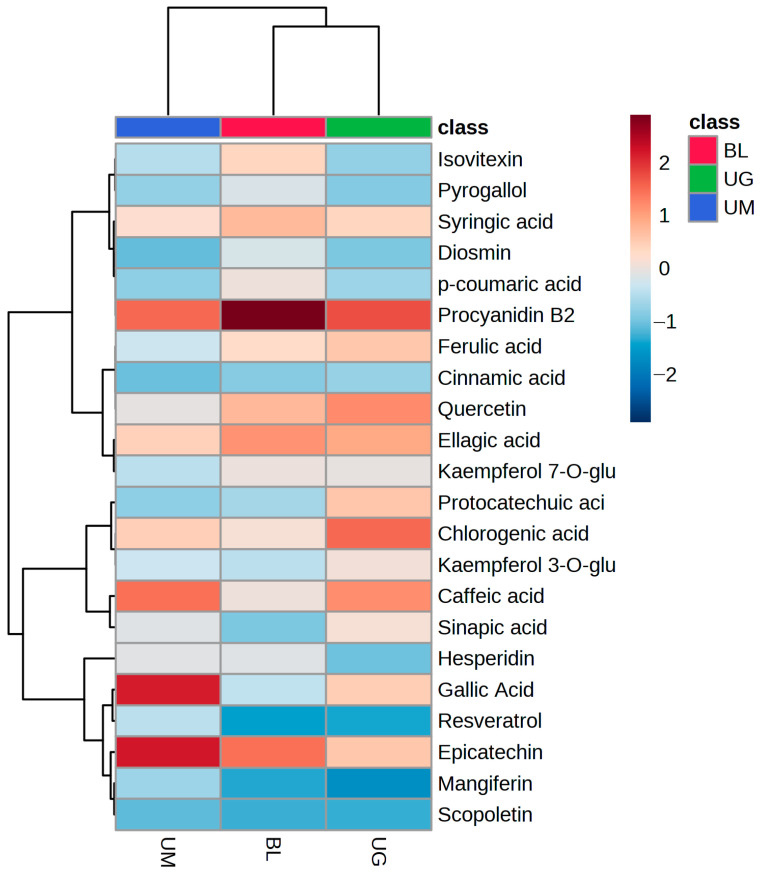
Pearson heatmap clustering of quantified phenolic compounds in wasted sour fruits.

At the same time, heatmap clustering was also conducted for the quantified phenolic compounds in these fruits (Figure 3). The heatmap shows that procyanidin B2 has the highest concentration in black lemon, while it is also quantified in unripe mangoes and grapes. Gallic acid, epicatechin, caffeic acid, chlorogenic acid, syringic acid, mangiferin, and ellagic acid have higher concentrations than other quantified compounds in unripe mangoes, while chlorogenic acid, procyanidin B2, caffeic acid, quercetin, ellagic acid, and ferulic acid were quantified in higher concentrations in unripe grapes than other compounds. Previously, gallic acid, chlorogenic acid, protocatechuic acid, vanillic acid, and ferulic acid in mango at different ripening stages were reported by Palafox-Carlos et al. [53].

### 2.7. Chemometric Analysis of Phenolic Compounds in Unripe Fruits

**Figure 4:**
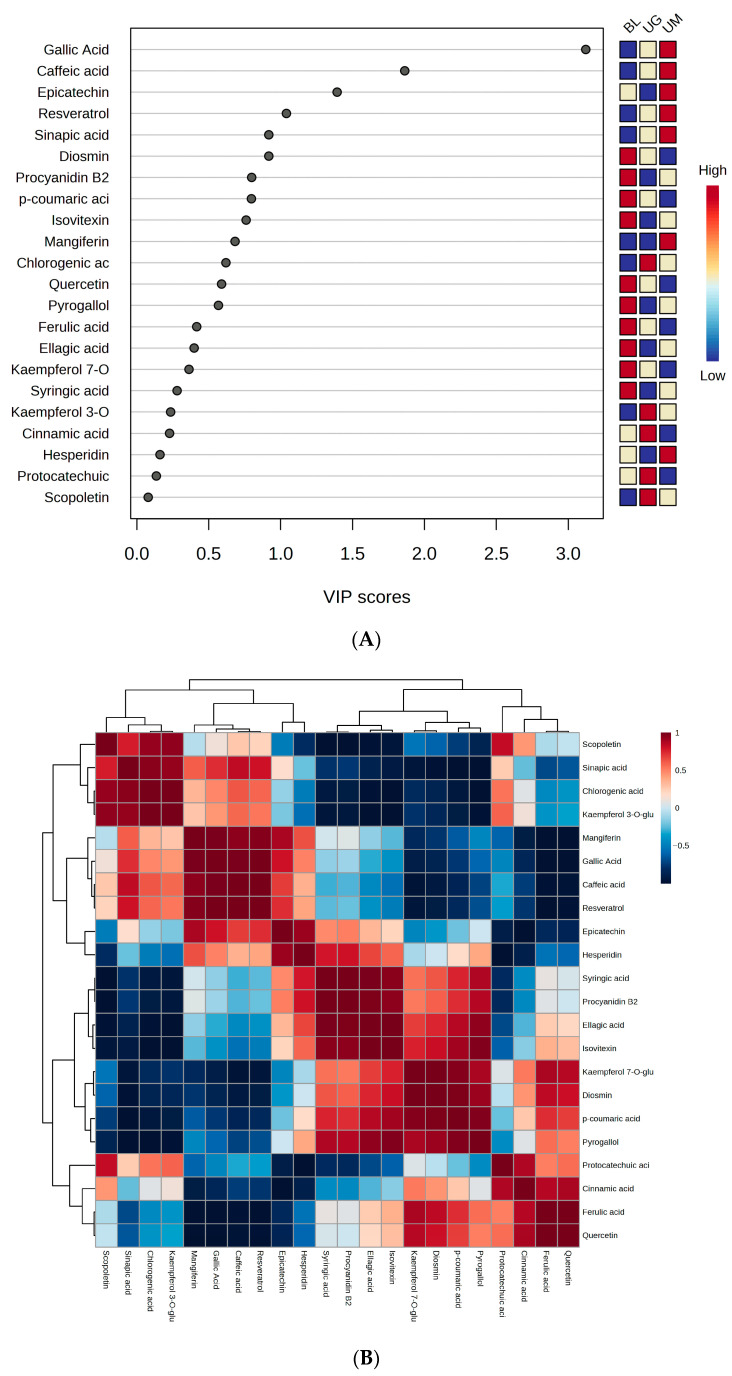

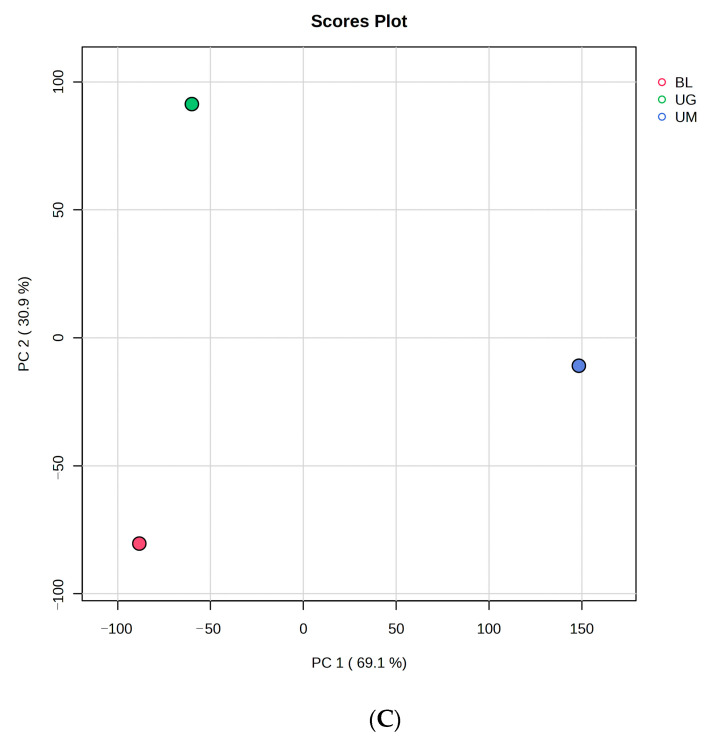
We computed partial-least-squares-discriminant-analysis derived variable’s importance in projection (VIP) score (**A**), Pearson correlation heatmap (**B**), and principal component analysis 2D scores plot (**C**) of quantified compounds in black lemon (BL), unripe grapes (UG) and unripe mangoes (UM).

Chemometrics analysis is a versatile science used to figure out the importance variables and observations in a complex dataset. We computed a partial-least-squares-discriminant-analysis derived variable’s importance in projection (VIP) score (4A), Pearson correlations heatmap (4B), and a principal component analysis 2D score plot (4C), as displayed in Figure 4. Figure 4A depicts that gallic acid, caffeic acid, epicatechin, resveratrol, sinapic acid, and diosmin are the biomarker phenolic compounds in the selected unripe sour fruits. Figure 4B illustrates that quercetin has the strongest correlation with ferulic acid and chlorogenic acid. The cell with dense red color shows the higher correlation between phenolic compounds. Figure 4C displays the importance of individual samples based on the concentration of phenolic compounds and depicts that unripe mangoes have the highest contribution of phenolic compounds which share almost 69%.

## 3. Materials and Methods

### 3.1. Materials

Analytical-grade chemicals were the only ones utilized in all the tests. Chemicals for identifying and characterizing compounds were supplied by Sigma Aldrich, located in Darmstadt, Germany. To estimate polyphenols and antioxidant capacity, the following materials were purchased from Sigma Aldrich (Castle Hill, NSW, Australia): potassium ferrocyanide (III), hydrated sodium acetate, trichloroacetic acid, vanillin, ammonium molybdate, sodium phosphate dibasic heptahydrate, Folin–Ciocalteu’s reagent, 2,2′-azino-bis 3-ethylbenzothiazoline-6-sulfonic acid (ABTS), ethylenediaminetetraacetic acid (EDTA), catechin, 2,2-diphenyl-1-picryl-hydrazyl-hydrate (DPPH), 2,4,6 tripyridyl-s-triazine (TPTZ), and 3-hydrobenzoic acid, as well as quercetin.

### 3.2. Sample Preparation and Method Optimization for Extraction of Phenolic Compounds

The samples of unripe mangoes, grapes, and black lemons were collected from the local market of Victoria, Australia. The unripe mangoes and grapes were collected in dried powder form, while the black lemon was collected in dried whole form. A laboratory grinder was used to grind the material into a fine powder. The process used to extract the phenolic compounds was as follows: Three separate extracts of the selected unripe sour fruits waste were prepared using one gram of powder and twenty milliliters of an 80% solvent (methanol) with 0.1 formic acid in Milli-Q water in triplicate. Samples were shaken for 16 h at 150 rpm and 4 °C in an orbital shaker (ZWYR-240) before being centrifuged. Following a 20 min centrifugation at 8000 rpm, the samples were separated into their supernatant and filtered through a 0.45 µm syringe filter. Prior to LC-MS/MS as well as spectrophotometric analysis, samples were stored at −20 °C for a maximum duration of fifteen days.

### 3.3. Quantification of Phenolic Contents in Unripe Fruits

#### 3.3.1. Determination of Total Phenolic Content

Using the previously published methodology by Ali et al. [46], the phenolic chemical profile of the samples was examined. First, 200 µL of distilled water was combined with 25 µL of the Folin–Ciocalteu reagent (25% *v*/*v*). After adding 25 µL of the sample extract, the mixture was incubated for 5 min at 27 °C. Finally, 25 µL of 10% *w*/*w* sodium carbonate was incorporated into the reaction mixture, and it was incubated for a further hour at 27 °C in the dark. The measured absorbance of the samples was 760 nm. A standard curve was created against gallic acid to quantify the total phenolic content, ranging from 0 to 200 µg/mL in methanol. Milligram gallic acid equivalents, or GAE units, were used to record the results per gram of sample.

#### 3.3.2. Total Flavonoid Content

A modified version of the procedure outlined by Ali et al. [64] was used to determine the flavonoid content of the samples. The TFC was determined using the aluminum chloride colorimetric technique in which 96-well plates were filled with an 80 µL sample extract, 80 µL AlCl_3_ solution, and 120 µL sodium acetate aqueous solution (50%). Following reaction mixture preparation, the prepared product was incubated for 2.5 h at 27 °C in the dark, and absorbance at 440 nm was measured using a spectrophotometer. A standard curve was created to determine flavonoid concentration, plotted against 0–50 µg/mL of quercetin in methanol. We expressed the results in milligram quercetin equivalents per gram of the sample unit.

#### 3.3.3. Total Condensed Tannin

Initially, 150 µL of a 4% vanillin solution and 25 µL of the sample mixture were combined. The mixture was then added to 25 µL of 32% H_2_SO_4_. The last sample was incubated for 15 min at 25 °C. After calculating the absorbance at 500 nm, the standard catechin curve (0–1000 µg/mL) was created. Data are expressed in mg CE/g.

### 3.4. Antioxidant Activities of Unripe Fruits

#### 3.4.1. ABTS Radical Scavenging Assay

The ABTS assay was carried out using the procedures outlined by Kiani et al. [65]. A mixture of 7 mM ABTS solution and 140 mM potassium persulfate solution was incubated for 16 h in the dark to create an ABTS^+^ solution. After diluting the solution with ethanol, the absorbance value at 734 nm was 0.70 ± 0.02. After adding 10 µL of sample extract and 290 µL of ABTS^+^ solution to a 96-well plate, it remained at room temperature for six minutes. At 734 nm, the absorbance was measured. To accomplish the measurement (mg AAE/g), a standard curve was generated against ascorbic acid concentrations ranging from 0 to 150 µg/mL in water.

#### 3.4.2. DPPH Radical Scavenging Assay

A triplicate DPPH assay was conducted for every sample using the modified protocol outlined by Kiani et al. [65]. To begin, a 96-well plate technique was used to combine 25 µL sample extracts with 275 µL of DPPH dye (0.1 M) in methanol. Following preparation, the reaction mixture was incubated for 30 min at 25 °C in a dark environment. The spectrophotometer was then used to record the result at 517 nm. A standard curve was created against ascorbic acid (0–50 µg/mL) in water to assess the radical scavenging capabilities. Ascorbic acid equivalents in milligrams (mg AAE/g) were used to record the observed results.

#### 3.4.3. Hydroxyl Radical Scavenging Assay

The procedure of Sharifi-Rad et al. [66] uses the Fenton-type reaction method to determine the ^•^OH-RSA (hydroxyl radical scavenging activity) of the extracts. In this procedure, 50 µL of sample extract was mixed with 50 µL of 6 mM FeSO_4_·7H_2_O and 50 µL of 30% hydrogen peroxide. The mixture was then incubated for 10 min at 25 °C. The solution was then treated with 50 µL of 6 mM 3-hydroxybenzoic acid, and the absorbance was measured at 510 nm. The results are presented in mg AAE/g. A standard curve was constructed using ascorbic acid concentrations ranging from 0 to 300 µg/mL.

#### 3.4.4. Fe^2+^ Chelating Activity (FICA)

Using this approach, 15 µL of the extract was combined with 85 µL of water, 50 µL of ferrozine (5 mM), and 50 µL of ferrous chloride (2 mM); later, the mixture was incubated at 25 °C for 10 min. The wavelength at which the absorbance was measured was 562 nm. The data are given as mg EDTA/g and were quantified using a standard curve created with EDTA at 0 to 50 µg/mL.

### 3.5. LC-MS/MS Characterization of Phenolic Compounds

The procedure previously reported by Ali et al. [47,67] was used to separate and identify phenolic compounds from plant samples. Using Agilent’s MassHunter Workstation Software (version B.06.00), located in Santa Clara, CA, USA, phytochemicals were extracted and identified. The untargeted phenolic metabolites of selected unripe mangoes, grapes, and black lemon were analyzed using an Agilent 6520 LC-ESI-Q-TOF-MS/MS (Accurate-Mass Q-TOF LC/MS) fitted with an Agilent HPLC 1200 series. A guard column (C18 ODS, 4.0 × 2.0 mm) shielded the Synergi 4 µm Hydro-RP 80 LC column (250 × 4.6 mm), which was used to screen the phenolic extracts. The column was manufactured by Phenomenex, located in Torrance, CA, USA. The following gradient was observed during the injection procedure of an aliquot of 10 µL from each phenolic extract: the flow rates of mobile phase B (0.1% formic acid in acetonitrile) and mobile phase A (0.1% formic acid in Milli-Q water) were 600 µL/min. Zero to ten minutes (10–20% B), ten to twenty minutes (20–25% B), twenty to thirty minutes (25–30% B), thirty to forty minutes (30–45% B), forty to fifty minutes (45–60% B), fifty to sixty-five minutes (60–90% B), sixty-five to sixty-seven minutes (90–100% B), sixty-seven to sixty-eight minutes (100–10% B), and sixty-eight to seventy minutes (10% B). Using the auto MS/MS mode, the following LC parameters were used: scan mode 50–1300 amu, capillary voltage (3500 V), nebulization 45 pressure, nitrogen gas flow rate (9 L/min) at 325 °C, and collision energies (10, 20, and 40 eV). Phenolic metabolites were identified and characterized using the Personal Compounds Database Library (PCDL) for metabolites and the Agilent MassHunter Workstation Software Quality Analysis (version B.06.00). This study used semi-quantification to analyze 23 phenolic compounds, with two runs of each sample. Additionally, 40 MS/MS spectra of commercial standards were collected in this experiment. LC-MS/MS, in conjunction with 26 commercial standards, were used to create equations [47]. Comprehensive help was provided from the Human Metabolome Database (HMDB) [68] for the identification of phenolic compounds.

### 3.6. Statistical Analysis

The analysis of variance (ANOVA), biplot analysis, and Pearson correlations were performed using Minitab (version 18.0, Minitab, LLC, State College, PA, USA) and XLSTAT-2019.1.3. We report phenolic content and biological activity results as mean + standard deviation. MetaboAnalyst 5.0 was used for chemometric analysis and heatmap clustering.

## 4. Conclusions

A total of 85 phenolic compounds (70 in black lemons, 49 in unripe grapes, and 68 in unripe mango) were identified, and 23 phenolic compounds were quantified using LC-ESI-QTOF-MS/MS. Unripe mangoes were measured as exhibiting the highest concentration of total phenolic content and antioxidant potential compared to unripe grapes and black lemon. This study confirmed that these unripe fruits are abundant with phenolic acids and flavonoids. There is notable variation in total phenolic and flavonoids recognized in unripe sour fruit waste. Procyanidin B2, gallic acid, epicatechin, caffeic acid, quercetin, and chlorogenic acid were measured as exhibiting higher concentrations in these selected unripe fruits. A positive correlation was found between phenolic contents and antioxidant activities of unripe fruits. The complementary profile of phenolic compounds in these unripe fruits makes them attractive, especially as dietary supplements in functional foods. The results of this research confirmed that unripe mangoes, grapes, and black lemons have vital polyphenols beneficial for human health.

## Figures and Tables

**Table 1:** Measurement of phenolic contents in unripe fruits.

Variables	Black Lemon	Unripe Mango	Unripe Grapes
TPC (mg GAE/g)	23.08 ± 2.28 ^b^	58.01 ± 6.37 ^a^	19.42 ± 1.16 ^c^
TFC (mg QE/g)	16.41 ± 1.02 ^b^	44.94 ± 5.02 ^a^	15.01 ± 1.12 ^b^
TCT (mg CE/g)	4.01 ± 0.32 ^b^	11.41 ± 0.91 ^a^	4.28 ± 0.62 ^b^

Total phenolic content (TPC), total flavonoid content (TFC), total condensed tannins (TCT), quercetin equivalent (QE), catechin equivalent (CE), and gallic acid equivalent (GAE). All values are significantly different (*p* < 0.05) in the same rows (^a–c^). All data are presented as mean (*n* = 3) with standard deviation (±).

**Table 2:** Quantification of antioxidant potential in unripe fruits.

Variables	Black Lemon	Unripe Mango	Unripe Grapes
DPPH (mg AAE/g)	32.53 ± 2.47 ^b^	114.27 ± 9.42 ^a^	23.71 ± 2.17 ^c^
ABTS (mg AAE/g)	69.25 ± 5.17 ^b^	134.12 ± 9.63 ^a^	53.44 ± 4.79 ^c^
FICA (mg EDTA/g)	14.24 ± 1.07 ^b^	33.16 ± 2.08 ^a^	11.49 ± 0.87 ^c^
OH-RSA (mg AAE/g)	34.16 ± 3.08 ^b^	57.31 ± 4.85 ^a^	19.57 ± 1.42 ^c^

The data are presented as mean ± standard deviation in triplicate (*n* = 3). Values in rows with superscript letters (^a–c^) are significantly different from each other (*p* < 0.05).

**Table 3:** Pearson’s correlation between phenolic contents and antioxidant activities of unripe fruits.

Variables	TPC	TFC	TCT	DPPH	ABTS	FICA
TFC	**1.000**					
TCT	0.993	**0.997**				
DPPH	**1.000**	**0.999**	0.993			
ABTS	0.995	0.990	0.977	0.995		
FICA	**1.000**	**0.997**	0.989	**1.000**	**0.998**	
OH-RSA	0.953	0.939	0.911	0.954	0.979	0.962

Values in bold are different from 0 with a significance level of alpha = 0.05.

**Table 4:** LC-MS/MS Characterization of phenolic compounds in unripe fruits.

No.	Name	Formula	RT	ESI +/−	Theoretical (*m*/*z*)	Observed (*m*/*z*)	Mass Error (ppm)	MS/MS	Samples
	Flavonoids								
	Anthocyanins								
1	Cyanidin 3-rhamnoside 5-glucoside	C_27_H_31_O_15_	20.698	[M]^+^	595.1706	596.1706	−0.50	449, 287	BL
2	Petunidin 3-rhamnoside 5-glucoside	C_28_H_33_O_16_	22.240	[M]^+^	625.1842	625.1839	−0.48	480, 317	BL
3	* Cyanidin	C_24_H_25_O_12_	26.649	[M]^+^	287	287	1.98	287	BL, UG, UM
	Flavanols								
4	* Epicatechin	C_15_H_14_O_6_	15.19	[M − H]^−^	289.0717	289.0711	−2.1	245, 205	UG, BL, UM
5	* Procyanidin B2	C_30_H_26_O_12_	19.321	[M − H]^−^	577.1351	577.1366	2.6	451, 425, 407, 289	UM, UG, BL
6	Procyanidin trimer C1	C_45_H_38_O_18_	20.808	[M + H]^+^	867.2131	867.2162	3.57	867	UG, UM
7	Procyanidin dimer B2	C_30_H_26_O_12_	21.056	** [M − H]^−^	579.1497	579.1529	5.53	579	UM, UG
8	(−)-Epigallocatechin 7-*O*-glucuronide	C_21_H_22_O_13_	36.003	[M + H]^+^	483.1133	483.1126	−1.45	483	UM, BL
	Flavanones								
9	Didymin	C_28_H_34_O_14_	13.090	[M + H]^+^	595.2022	595.2032	1.68	577, 287	BL, UM
10	6-Geranylnaringenin	C_25_H_28_O_5_	15.226	[M − H]^−^	407.1864	407.1890	6.39	287, 243, 159, 119	BL
11	Hesperidin	C_28_H_34_O_15_	22.590	[M + H]^+^	611.1971	611.1974	0.49	303	UG, BL, UM
12	Hesperetin 3′-*O*-glucuronide	C_22_H_22_O_12_	23.133	[M + H]^+^	479.1184	479.1199	3.13	301	UG
	Flavones								
13	Nobiletin	C_21_H_22_O_8_	3.726	** [M − H]^−^	401.1242	401.1225	−4.24	401	UG, BL
14	Apigenin 6-*C*-glucoside	C_21_H_20_O_10_	4.175	** [M − H]^−^	431.0983	431.0990	1.62	269	UG, BL, UM
15	Apigenin 7-*O*-glucoside	C_21_H_24_O_9_	6.652	[M − H]^−^	419.1347	419.1327	−4.77	417	UG, UM
16	Apigenin 6,8-di-*C*-glucoside	C_27_H_30_O_15_	16.818	[M + H]^+^	595.1658	595.1691	5.54	595	BL, UM
17	6-Hydroxyluteolin 7-*O*-rhamnoside	C_21_H_20_O_11_	21.552	[M + H]^+^	449.1079	449.1109	6.68	303, 285	UG
18	* Diosmin	C_28_H_32_O_15_	22.666	** [M − H]^−^	609.1814	609.1868	8.86	301	UM, BL, UG
	Flavonols								
19	Kaempferol 3,7,4′-*O*-triglucoside	C_33_H_40_O_21_	4.530	[M + H]^+^	773.2135	773.2150	1.94	773	UM, BL
20	Isorhamnetin 3-*O*-glucuronide	C_22_H_20_O_13_	4.608	[M + H]^+^	493.0977	493.0979	0.41	493	UM, BL
21	Kaempferol 7-*O*-glucoside	C_21_H_19_O_11_	16.662	[M + H]^+^	448.1000	448.1041	9.15	448	BL, UM
22	Myricetin	C_15_H_10_O_8_	16.934	[M + H]^+^	319.0449	319.0451	0.63	319	UG, BL, UM
23	Dihydroquercetin	C_15_H_12_O_7_	22.220	[M + H]^+^	305.0656	305.0668	3.93	305	UG
24	Kaempferol 3-*O*-glucuronide	C_21_H_18_O_12_	23.133	[M + H]^+^	463.0871	463.0899	6.05	463	UG, UM
25	Dihydromyricetin 3-*O*-rhamnoside	C_21_H_22_O_12_	24.926	[M + H]^+^	467.1184	467.1174	−2.14	467	BL, UG
26	* Quercetin	C_15_H_10_O_7_	28.760	[M + H]^+^	303.0499	303.0525	8.58	285, 169	UG, UM, BL
	Isoflavonoids								
27	6″-*O*-Acetylglycitin	C_24_H_24_O_11_	4.055	[M + H]^+^	489.1392	489.1380	−2.45	489	BL, UM
28	6″-*O*-Acetylgenistin	C_23_H_22_O_11_	4.737	[M + H]^+^	475.1235	475.1246	2.32	475	BL, UM
29	4′,7-Dihydroxyisoflavan	C_15_H_14_O_3_	12.000	[M − H]^−^	241.0870	241.0877	2.90	241	BL, UM
30	Violanone	C_17_H_16_O_6_	26.037	[M + H]^+^	317.1020	317.1034	4.41	317	UG, UM
31	Dihydroformononetin	C_16_H_14_O_4_	30.090	[M + H]^+^	271.0965	271.0969	1.48	271	UG, BL
32	3′-*O*-Methylequol	C_16_H_16_O_4_	33.387	[M + H]^+^	273.1122	273.1124	0.73	273	UM, BL
33	Dihydrobiochanin A	C_16_H_14_O_5_	49.864	[M + H]^+^	287.0914	287.0920	2.09	269, 203, 201, 175	UM, BL
	Chalcones								
34	Phloridzin	C_21_H_24_O_10_	7.307	** [M − H]^−^	435.1297	435.1294	−0.69	435	BL, UG
35	Phloretin 2′-*O*-glucuronide	C_21_H_22_O_11_	22.220	[M + H]^+^	451.1235	451.1256	4.66	275	UM, UG
	Stilbenes								
36	4′-Hydroxy-3,4,5-trimethoxystilbene	C_17_H_18_O_4_	4.150	[M + H]^+^	287.1278	287.1272	−2.09	287	UM, UG
37	Piceatannol 3-*O*-glucoside	C_20_H_22_O_9_	6.120	** [M − H]^−^	405.1191	405.1186	−1.23	245	UG, BL, UM
38	Dihydroresveratrol	C_14_H_14_O_3_	11.128	** [M − H]^−^	229.0870	229.0872	0.87	229, 81	UM, BL
39	Resveratrol	C_14_H_12_O_3_	21.552	[M + H]^+^	229.0859	229.0876	7.42	211, 167, 127	UM, UG
40	Piceatannol	C_14_H_12_O_4_	54.463	[M + H]^+^	245.0809	245.0823	5.71	245	BL, UM
	Lignans								
41	7-Hydroxymatairesinol	C_20_H_22_O_7_	4.649	[M + H]^+^	375.1439	375.1437	−0.53	375	UM, BL
42	Schisantherin A	C_30_H_32_O_9_	5.663	[M − H]^−^	535.1973	535.1946	−5.04	535	BL, UM
43	Lariciresinol	C_20_H_24_O_6_	6.147	** [M − H]^−^	359.1500	359.1515	4.18	329, 192, 178, 175	BL, UG
44	2-Hydroxyenterolactone	C_18_H_18_O_5_	13.283	** [M − H]^−^	313.1081	313.1083	0.64	255	UG, BL, UM
45	2-Hydroxyenterodiol	C_18_H_22_O_5_	13.698	[M − H]^−^	317.1394	317.1378	−5.05	299, 287, 269, 257	UG, BL
46	Schisanhenol	C_23_H_30_O_6_	14.084	[M − H]^−^	401.1969	401.1975	1.50	401	BL, UM
47	Secoisolariciresinol	C_20_H_26_O_6_	15.100	[M − H]^−^	361.1656	361.1635	−5.81	346, 315, 223, 165	UM, UG
	Phenolic Acids								
	Hydroxybenzoic acids								
48	Protocatechuic acid 4-*O*-glucoside	C_13_H_16_O_9_	4.037	** [M − H]^−^	315.0721	315.0746	7.9	153	BL, UG, UM
49	* Gallic acid	C_7_H_6_O_5_	7.388	** [M − H]^−^	169.0142	169.0144	0.6	125	BL, UM, UG
50	Ellagic acid	C_14_H_6_O_8_	7.414	** [M − H]^−^	300.9990	300.9990	0.0	284, 257	BL, UM, UG
51	3-*O*-Methylgallic acid	C_8_H_8_O_5_	11.584	** [M − H]^−^	183.0299	183.0299	0.0	169	BL, UM
52	Paeoniflorin	C_23_H_28_O_11_	13.732	[M + H]^+^	481.1705	481.1744	8.1	481	BL, UM
53	* Protocatechuic acid	C_7_H_6_O_4_	15.860	** [M − H]^−^	153.0193	153.0195	1.3	109	BL, UM, UG
54	Gallic acid 3-monogallate	C_14_H_10_O_9_	15.993	** [M − H]^−^	323.0398	323.0381	−5.5	169, 125	BL, UM
55	*p*-Hydroxybenzoic acid	C_7_H_6_O_3_	16.818	** [M − H]^−^	137.0234	137.0243	−4.3	93	UG, BL, UM
56	* Syringic acid	C_9_H_10_O_5_	20.168	** [M − H]^−^	197.0455	197.0465	5.075	182, 153	UM, UG, BL
57	* Benzoic acid	C_7_H_6_O_2_	21.713	** [M − H]^−^	121.0295	121.0294	−0.8	103, 93	UG, UM, BL
	Hydroxycinnamic acids								
58	Cinnamoyl glucose	C_15_H_18_O_7_	9.676	** [M − H]^−^	311.1125	311.1114	−3.5	311	BL, UM
59	Caffeic acid 4-*O*-glucuronide	C_15_H_16_O_10_	9.734	** [M − H]^−^	355.0671	355.0653	−5.0695	179	UM, BL, UG
60	* Chlorogenic acid	C_16_H_18_O_9_	13.294	** [M − H]−	353.0878	353.0874	−1.1	191, 179, 161	BL, U, UM
61	* p-Coumaric acid	C_9_H_8_O_3_	14.433	** [M − H]^−^	163.0400	163.0411	6.7	119	BL, UG, UM
62	*p*-Coumaric acid 4-*O*-glucoside	C_15_H_18_O_8_	16.629	** [M − H]^−^	325.0929	325.0936	2.2	163	BL, UM
63	* Caffeic acid	C_9_H_8_O_4_	17.639	** [M − H]^−^	179.0350	179.0351	0.6	135	UG, UM, BL
64	* Cinnamic acid	C_9_H_8_O_2_	18.021	** [M − H]^−^	147.0451	147.0441	−6.8	103	UG, BL, UM
65	Dihydroferulic acid	C_10_H_12_O_4_	18.986	[M + H]^+^	197.0809	197.0818	4.57	197	UM, BL
66	* Ferulic acid	C_10_H_10_O_4_	21.124	** [M − H]^−^	193.0506	193.0508	1.036	178, 149, 134	UM, UG, BL
67	1,2,2′-Triferuloylgentiobiose	C_42_H_46_O_20_	22.826	[M + H]^+^	871.2655	871.2631	−2.75	871	UM, BL
68	* Sinapic acid	C_11_H_12_O_5_	24.062	[M − H]^−^	223.0612	223.0618	2.7	193, 179, 149, 134	UM, BL
69	Verbascoside A	C_31_H_40_O_16_	28.316	[M + H]^+^	669.2389	669.2399	1.49	669	UM, BL
70	* Rosmarinic acid	C_18_H_16_O_8_	35.023	** [M − H]^−^	361.0918	361.0906	−3.32	197, 179	BL
71	*p*-Coumaroyl glycolic acid	C_11_H_10_O_5_	52.416	[M + H]^+^	223.0601	223.0615	6.28	223	BL, UM
	Other compounds								
72	3,4-DHPEA-EDA	C_17_H_20_O_6_	4.297	[M − H]^−^	319.1187	319.1191	1.25	275, 195	UM, BL
73	* Pyrogallol	C_6_H_6_O_3_	9.783	[M + H]^+^	127.0390	127.0395	3.94	127	BL, UM
74	*p*-HPEA-AC	C_10_H_12_O_3_	12.551	[M − H]^−^	179.0713	179.0702	−6.14	137, 119	UG, BL
75	Quinic Acid	C_7_H_12_O_6_	13.178	[M − H]^−^	191.0561	191.0572	5.76	171, 127, 85	UG, UM, BL
76	* Mangiferin	C_19_H_18_O_11_	13.992	[M − H]^−^	421.0776	421.0797	4.9872	331, 301, 259	UM
77	Catechol	C_6_H_6_O_2_	14.968	[M + H]^+^	111.0441	111.0449	7.20	111	UM, UG, BL
78	Mangiferin 6′-gallate	C_26_H_22_O_15_	16.163	[M − H]^−^	573.0886	573.0898	2.0939	421	UM
79	Mellein	C_10_H_10_O_3_	17.370	** [M − H]^−^	177.0557	177.0555	−1.13	133	UG, BL
80	*p*-Anisaldehyde	C_8_H_8_O_2_	18.986	[M + H]^+^	137.0597	137.0600	2.19	122, 109	UM, UG, BL
81	Carvacrol	C_10_H_14_O	45.040	[M + H]^+^	151.1118	151.1125	4.63	151	UM, BL
82	Esculetin	C_9_H_6_O_4_	47.592	[M + H]^+^	179.0339	179.0347	4.47	179	UM, BL
83	Coumarin	C_9_H_6_O_2_	50.738	[M + H]^+^	147.0441	147.0443	1.36	103, 91	UG, BL
84	4-Hydroxycoumarin	C_9_H_6_O_3_	52.252	[M + H]^+^	163.0390	163.0398	4.91	163	BL, UG
85	Scopoletin	C_10_H_8_O_4_	54.433	[M + H]^+^	193.0496	193.0505	4.66	193	UM, BL, UG

* = compounds identified with pure standards; ** = compounds detected in both ESI +/−. Black lemon (BL), Unripe Mango (UM), and Unripe grapes (UG).

**Table 5:** Targeted quantification/semi-quantification of phenolic compounds (μg/g).

No.	Compounds	Unripe Mango	Back Lemon	Unripe Grapes
1	Gallic Acid	221.17 ± 9.69	56.91 ± 1.32	86.43 ± 7.42
2	Protocatechuic acid	38.56 ± 1.02	45.71 ± 3.21	89.21 ± 5.42
3	Ellagic acid	113.93 ± 8.31	134.91 ± 11.42	101.25 ± 7.61
4	Caffeic acid	177.31 ± 5.01	79.28 ± 5.02	111.47 ± 6.16
5	Cinnamic acid	22.14 ± 1.34	34.13 ± 3.37	42.22 ± 3.67
6	*p*-coumaric acid	37.41 ± 2.24	79.34 ± 4.25	44.91 ± 2.19
7	Ferulic acid	69.51 ± 5.69	91.43 ± 7.38	88.54 ± 5.61
8	Chlorogenic acid	115.98 ± 8.79	83.37 ± 4.49	123.57 ± 9.31
9	Syringic acid	98.68 ± 9.36	113.42 ± 9.01	82.45 ± 6.13
10	Sinapic acid	79.64 ± 5.23	31.34 ± 2.18	73.12 ± 5.35
11	Kaempferol 7-*O*-glucoside	59.32 ± 4.43	78.42 ± 5.23	67.48 ± 5.21
12	Kaempferol 3-*O*-glucuronide	67.51 ± 4.73	55.12 ± 5.04	71.51 ± 3.65
13	Quercetin	83.15 ± 6.29	114.17 ± 9.05	111.92 ± 7.32
14	Epicatechin	224.31 ± 13.41	151.02 ± 6.17	88.52 ± 6.02
15	Isovitexin	56.64 ± 4.35	96.64 ± 7.39	41.36 ± 2.26
16	Diosmin	19.21 ± 1.57	67.51 ± 5.07	36.08 ± 3.19
17	Procyanidin B2	182.12 ± 9.34	224.15 ± 11.34	131.18 ± 10.06
18	Hesperidin	79.65 ± 7.35	71.16 ± 6.01	32.73 ± 2.08
19	Pyrogallol	39.47 ± 3.07	69.34 ± 4.27	37.73 ± 2.19
20	Mangiferin	44.93 ± 3.67	NQ	NQ
21	Resveratrol	58.62 ± 3.39	NQ	19.41 ± 1.54
22	Scopoletin	18.12 ± 1.36	13.95 ± 1.24	23.03 ± 1.07

Not quantified (NQ).

## Data Availability

Data are contained within the article.
